# Zeolite catalyzed solvent-free one-pot synthesis of dihydropyrimidin-2(1*H*)-ones – A practical synthesis of monastrol

**DOI:** 10.3762/bjoc.5.4

**Published:** 2009-02-04

**Authors:** Mukund G Kulkarni, Sanjay W Chavhan, Mahadev P Shinde, Dnyaneshwar D Gaikwad, Ajit S Borhade, Attrimuni P Dhondge, Yunnus B Shaikh, Vijay B Ningdale, Mayur P Desai, Deekshaputra R Birhade

**Affiliations:** 1 Department of Chemistry, University of Pune, Ganeshkhind, Pune 411007, Maharashtra, India

**Keywords:** Biginelli reaction, DHPMs, neat, MCR, zeolite

## Abstract

A zeolite-catalyzed, simple, one-pot, solvent-free, cost effective, and environmentally benign process for the synthesis of dihydropyrimidones is described. This reaction is scaleable to multigram scale and the catalyst is recyclable. This methodology has resulted in an efficient synthesis of monastrol, a potent inhibitor of kinesin Eg5.

## Introduction

The Biginelli reaction is a well-known multicomponent reaction involving a one-pot cyclocondensation of an aldehyde, β-ketoester and urea/thiourea [1–3]. Multicomponent reactions (MCRs) have recently gained tremendous importance in organic and medicinal chemistry. The main contributing factors are the high atom economy, wide application in combinatorial chemistry and diversity-oriented synthesis [4–10]. In general, the dihydropyrimidones (DHPMs) are known for their diverse important biological activities like antiviral, antitumor, antibacterial and antiinflammatory properties [11–13].

The first report of the Biginelli reaction dates back to 1893. This involved a very simple and straightforward procedure for the synthesis of DHPMs. A one-pot cyclocondensation of ethyl acetoacetate, an aromatic aldehyde and urea under strongly acidic conditions furnished the corresponding DHPMs [1]. Unfortunately this method led to low to moderate yields of the desired DHPMs, particularly when substituted aromatic or aliphatic aldehydes and thiourea were employed [14–20].

To overcome this problem, various homogeneous as well as heterogeneous catalysts have been utilized. Typical examples of the various homogeneous catalysts employed are polyphosphate ester [18], LaCl_3_·7H_2_O [21] and LiClO_4_ [22]. Recently Lewis acid catalyzed Biginelli reactions have been extensively reported in the literature. This involved the use of Lewis acids like Yb(OTf)_3_ [23], CuCl_2_ [24], Mn(OAc)_3_ [25], Bi(OTf)_3_ [26], CeCl_3_·7H_2_O [27], Cu(OTf)_2_ [28], FeCl_3_ [29], BF_3_·Et_2_O/Cu(OAc)_2_ [30], ZrCl_4_ [31], polymer supported ytterbium reagents [32], TaBr_5_ [33], ZrCl_4_ or ZrOCl_2_ [34]. Baker’s yeast has also been used recently for this purpose [35]. The Brønsted acid mediated Biginelli reactions using *p*-TSA [36], H_2_SO_4_/SiO_2_ [37], and KHSO_4_ [38] are known in the literature. The heterogeneous catalysts used in this reaction involve the use of KSF (montmorillonite) [39], bentonitic clay [40], and zeolites like HZSM-5, Hy, MCM–41 [41]. The limitations in using the above mentioned catalysts were elevated reaction temperatures, solvent mediated reactions and moderate yields of the products. Apart from these, the heterogeneous catalysts were required in stoichiometric amounts. Furthermore, when aliphatic aldehydes and thiourea were used low yields of DHPMs were realized.

## Results and Discussion

**Scheme 1:**
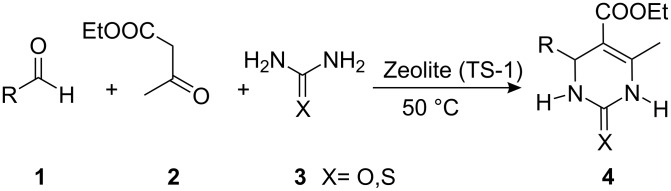
General procedure for the synthesis of DHPMs.

We herein report a one-pot synthesis of DHPMs using a catalytic amount of zeolite under solvent-free conditions. In our quest to bring about the Biginelli reaction, we attempted the reaction using zeolite under solvent-free conditions. The well-known titanium silicate TS-1 (also known as titanium silicalite) [42] is a widely used heterogeneous catalyst. Therefore we decided to explore its suitability in the Biginelli reaction. For this purpose, a mixture of an aldehyde **1** (1 mmol), ethyl acetoacetate (**2**, 1 mmol), urea **3** (1.5 mmol) and TS-1 (10 wt %) was stirred at room temperature for 10 min (TLC check). The solid product **4** obtained was extracted in hot ethanol and on concentrating the extract the product was obtained albeit in low yield (25%). Even after stirring the reaction mixture for prolonged time, no substantial increase in the yield was observed. Hence the reaction mixture was heated at 50 °C for 10 min (TLC check). Following the above workup procedure, the product was obtained in 98% yield (Scheme 1).

Next we explored the effect of the amount of catalyst on the yield of the product obtained in the reaction. After several runs using diminishing quantities of the catalyst it was found that there is no effect on the product yield even when the catalyst was used to the extent of 2 wt %. So all the further reactions were conducted using 2 wt % of catalyst. Under the present reaction conditions, even on using excess of ethyl acetoacetate (2 mmol), no side product arising through Knoevenagel condensation [41] was observed.

This methodology was effective for aliphatic as well as aromatic aldehydes in the preparation of dihydropyrimidones (**4a**–**4l**) with uniformly high yields (Table 1).

**Table 1:** Titanium silicate (TS-1) catalyzed synthesis of DHPMs.

Prod No.	R	X	Time (min)	Yield (%)

**4a**	Ph	O	10	98
**4b**	CH_3_	O	10	93
**4c**	CH_3_-CH_2_	O	12	97
**4d**	CH_3_-CH_2_-CH_2_	O	15	97
**4e**	4-(OH)-C_6_H_4_	O	30	94
**4f**	3-(OH)-C_6_H_4_	O	25	95
**4g**	2-(NO_2_)-C_6_H_4_	O	35	90
**4h**	4-(Cl)-C_6_H_4_	O	20	96
**4I**	Ph-CH=CH^a^	O	10	96
**4j**	3,4-di-(OMe)C_6_H_3_	O	18	96
**4k**	2-furyl	O	12	95
**4l**	5-methyl-2-furyl	O	15	95
**4m**	CH_3_	S	20	95
**4n**	3-(OH)-C_6_H_4_	S	33	94
**4o**	4-(Cl)-C_6_H_4_	S	20	95
**4p**	Ph-CH=CH	S	14	95

^a^commercially available cinnamic aldehyde was used.

This catalyst also worked well even with an acid-sensitive aldehyde such as furfural (**4k**–**4l**) without leading to the formation of any side products. The structural variations in the aldehydes employed in the reaction has no effect on the yield of the reaction, which is uniformly high. Significantly, a variety of sensitive functional groups like the NO_2_, Cl, OH, OCH_3_ and conjugated double bonds affect neither the course nor the yield of the reaction.

**Figure 1:**
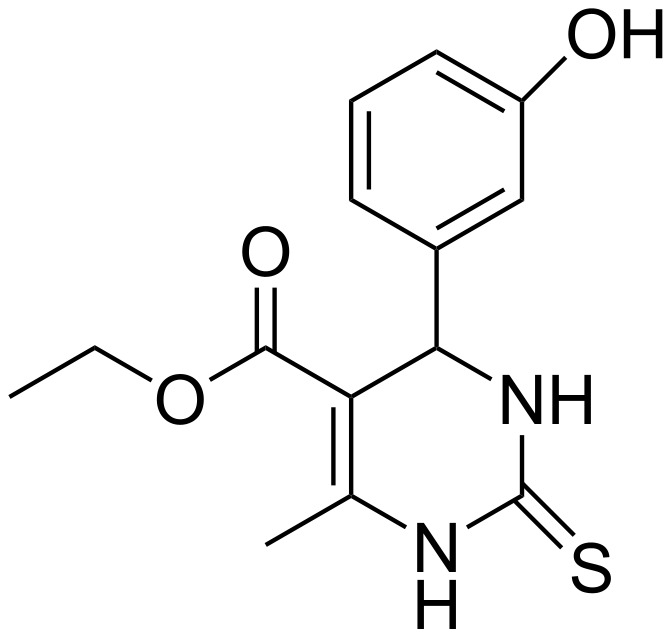
Monastrol (**4n**).

The scope of the reaction was further expanded when the reaction was carried out successfully using thiourea to provide the corresponding dihydropyrimidin-2(1*H*)-thiones (**4m**–**4p**). These thiones are also of much interest with regard to biological activity. One of the well-known examples of dihydropyrimidin-2-(1*H*)-thiones is the compound monastrol (**4n**), a potent inhibitor of kinesin Eg5 (Figure 1).

We could achieve the synthesis of this compound in one step using 3-hydroxybenzaldehyde, thiourea, ethyl acetoacetate and TS-1 under the above mentioned reaction conditions. Monastrol was obtained in 94% yield.

The most important and salient feature of the present reaction is the recyclability of the catalyst and the scaleability of the reaction. It was observed that the catalyst could be reused at least seven times. Use of the recycled catalyst in the reaction had no effect either on the yield of the product or the quality of the product. Moreover no side products were observed in these reactions. Furthermore, the reaction can be scaled up to a multigram scale. This was demonstrated by preparing 11.2 g of monastrol starting with 5.0 g of 3-hydroxybenzaldehyde. Thus an efficient one-step, solvent-free synthesis of DHPMs was achieved in very good yields.

## Conclusion

In conclusion, we have developed a practical methodology for the synthesis of dihydropyrimidones, which has been successfully extended to the multigram synthesis of Monastrol.

## Experimental

### Typical procedure for the synthesis of DHPMs

#### 5-Ethoxycarbonyl-4-phenyl-6-methyl-3,4-dihydropyrimidin-2(1*H*)-one (**4a**)

A mixture of benzaldehyde (0.50 g, 4.71 mmol), ethyl acetoacetate (0.613 g, 4.71 mmol), urea (0.424 g, 7.07 mmol), and catalyst TS-1 (0.01 g, 2 wt % in relation to amount of benzaldehyde used) was heated at 50 °C for 10 min (TLC check). The reaction mixture after cooling to room temperature was poured into crushed ice and stirred for 5–10 min. The solid separated was filtered and washed with ice-cold water. To separate the catalyst from the product, the mixture was treated with hot ethanol and filtered. The residue, being the catalyst, was dried and reused. The filtrate on concentration afforded the product, which was found to be sufficiently pure to obtain analytical data.

IR (KBr): 1606.6, 1647.1, 1664.5, 3215.1, 3319.3 cm ^−1^. ^1^H NMR (300 MHz, DMSO-*d*_6_): δ 1.10 (t, *J* = 7.1 Hz, 3H), 2.24 (s, 3H), 3.98 (q, *J* = 7.1 Hz, 2H), 5.13 (s, 1H), 7.30 (bs, 5H), 7.74 (s, 1H), 9.19 (s, 1H). ^13^C NMR (75 MHz, DMSO-*d*_6_): δ 14.1, 17.8, 53.9, 59.1, 99.1, 126.0, 127.1, 128.2, 142.4, 148.1, 152.0, 165.1. (EI, 70 eV): *m/z* 42, 44, 60, 75, 138, 182, 265.

## Supporting Information

File 1 ^13^C NMR spectra of compounds **4a**–**4p**

File 2 Experimental procedures for compounds **4a**–**4p**
