# Supplementary material for: Zeolite catalyzed solvent-free one-pot synthesis of dihydropyrimidin-2(1H)-ones – A practical synthesis of monastrol
Source: Beilstein J Org Chem. 2009 Feb 4;5:4. doi: 10.3762/bjoc.5.4 (PMC2649439; doi:10.3762/bjoc.5.4)
Supplement: File 1 — 13C NMR spectra of compounds 4a–4p [file Beilstein_J_Org_Chem-05-04-s001.doc]

# Zeolite catalyzed solvent-free one-pot synthesis of dihydropyrimidin-2(1*H*)-ones – A practical synthesis of monastrol

Mukund G. Kulkarni[[1]](#endnote-2)*, Sanjay W. Chavhan, Mahadev P. Shinde, Dnyaneshwar D. Gaikwad, Ajit S. Borhade, Attrimuni P. Dhondge, Yunnus B. Shaikh, Vijay B. Ningdale, Mayur P. Desai and Deekshaputra R. Birhade

Address: Department of Chemistry, University of Pune, Ganeshkhind, Pune 411007, Maharashtra, India

Email: Mukund G. Kulkarni - mgkulkarni@chem.unipune.ernet.in

* Corresponding author

## Supporting Information

^13^C NMR spectra of compounds **4a**–**4p**


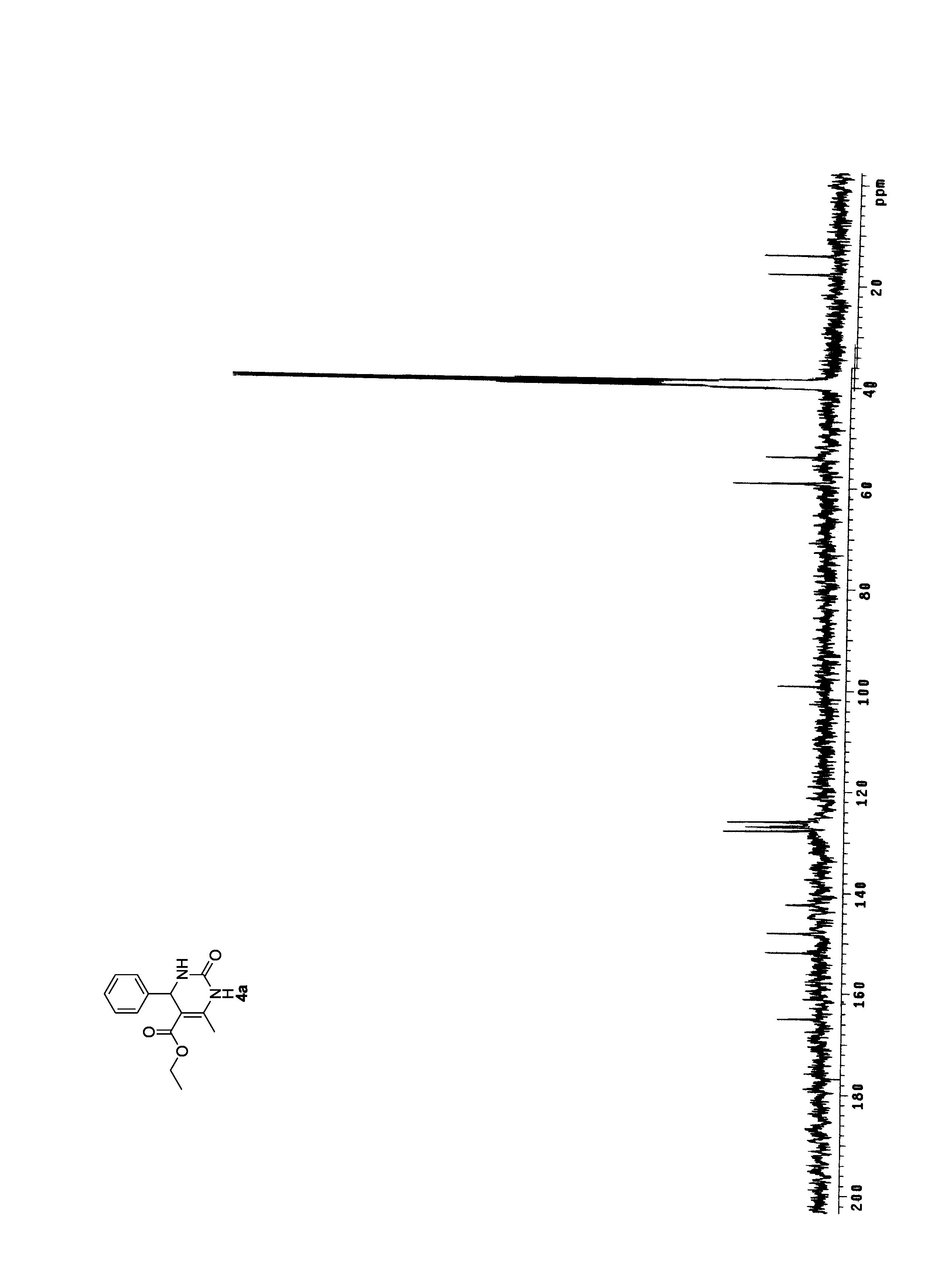


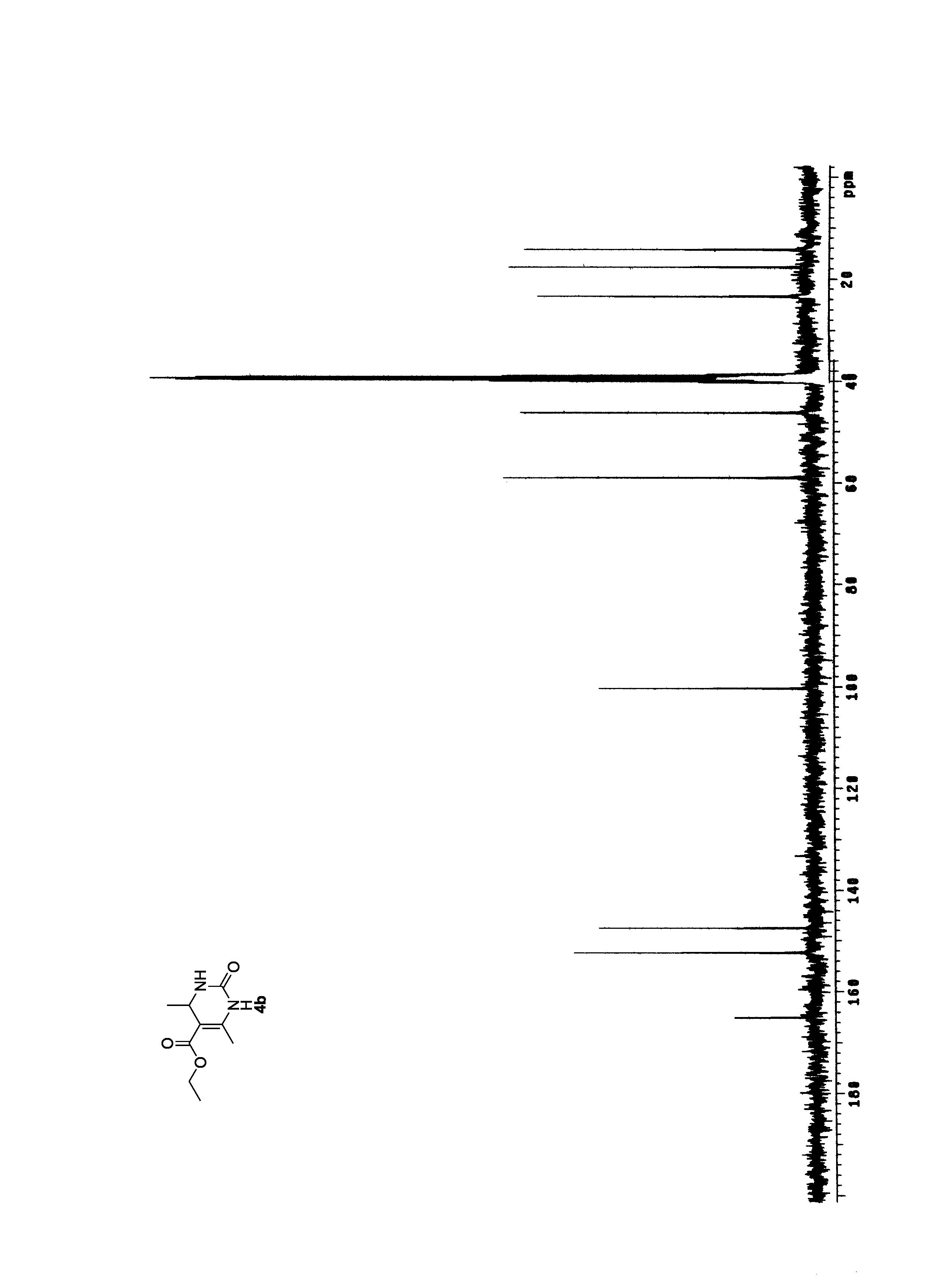


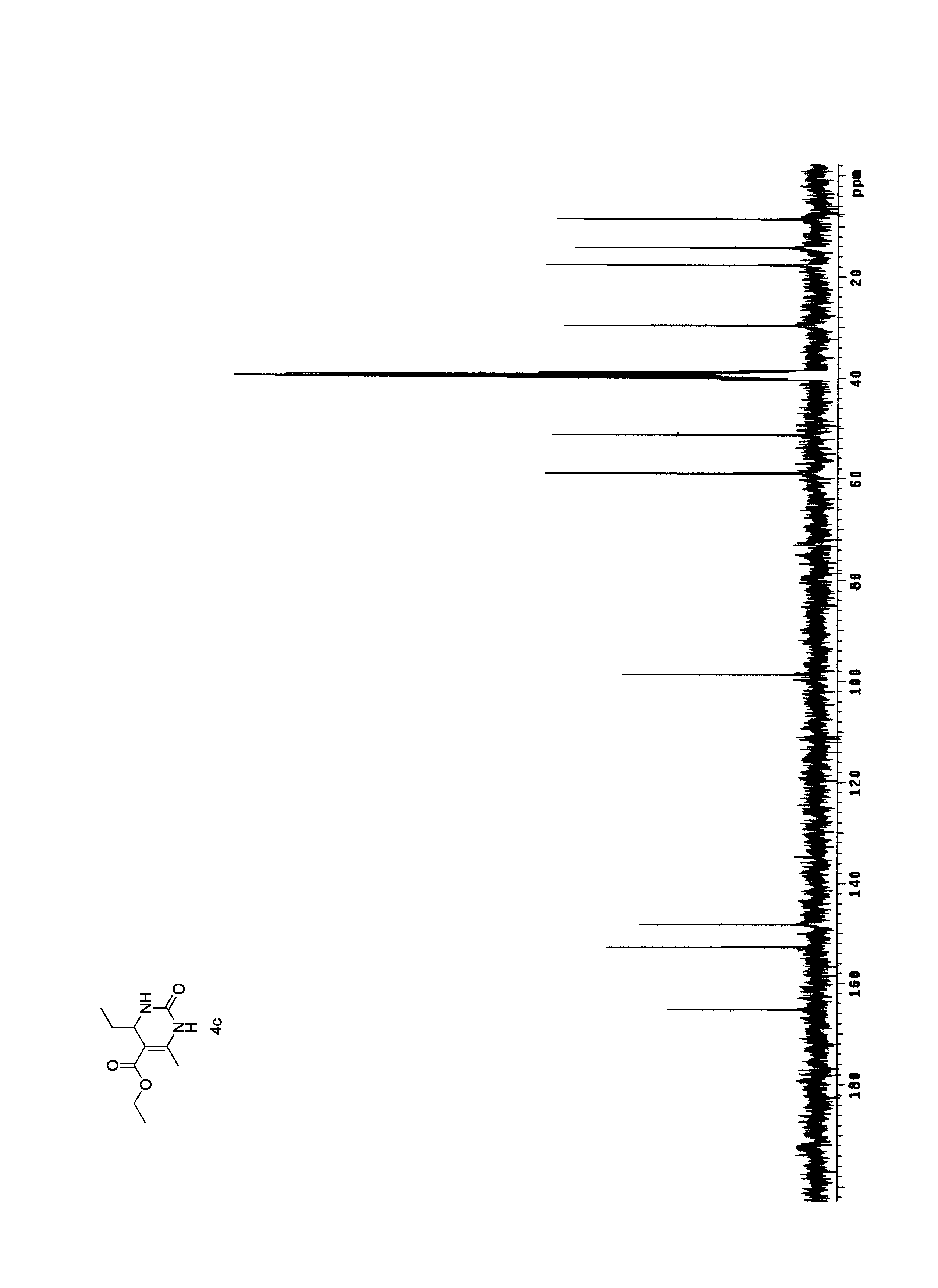


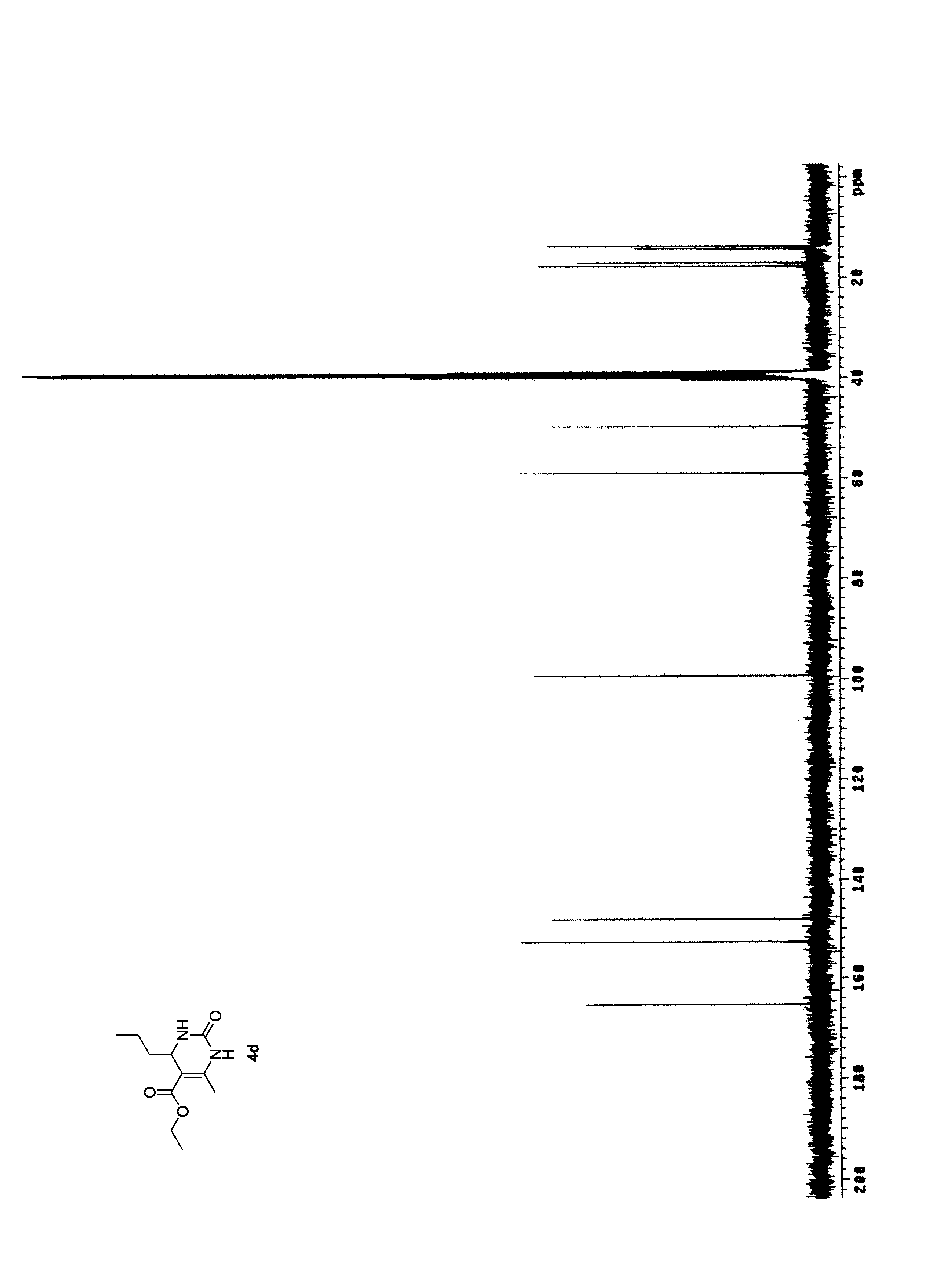


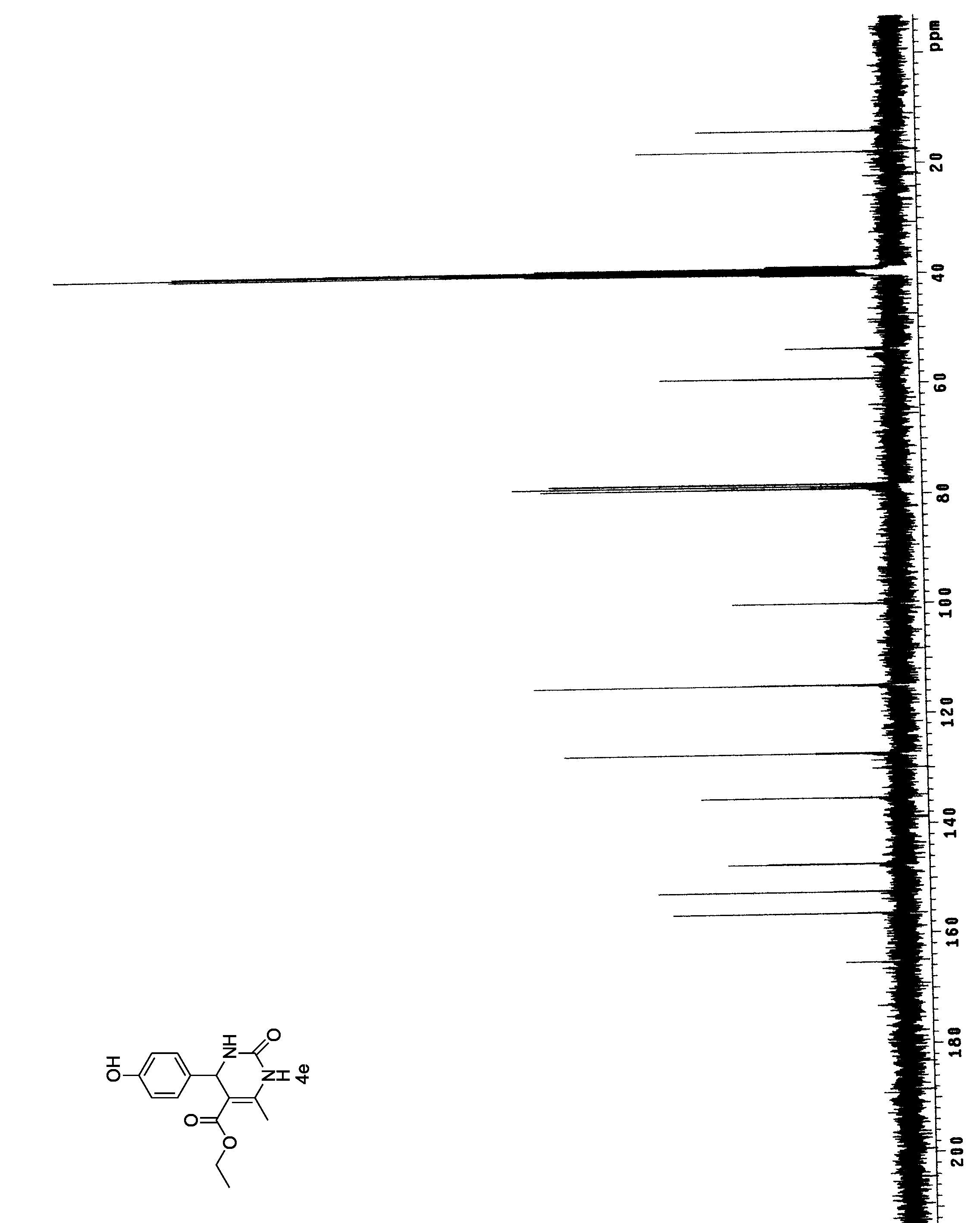


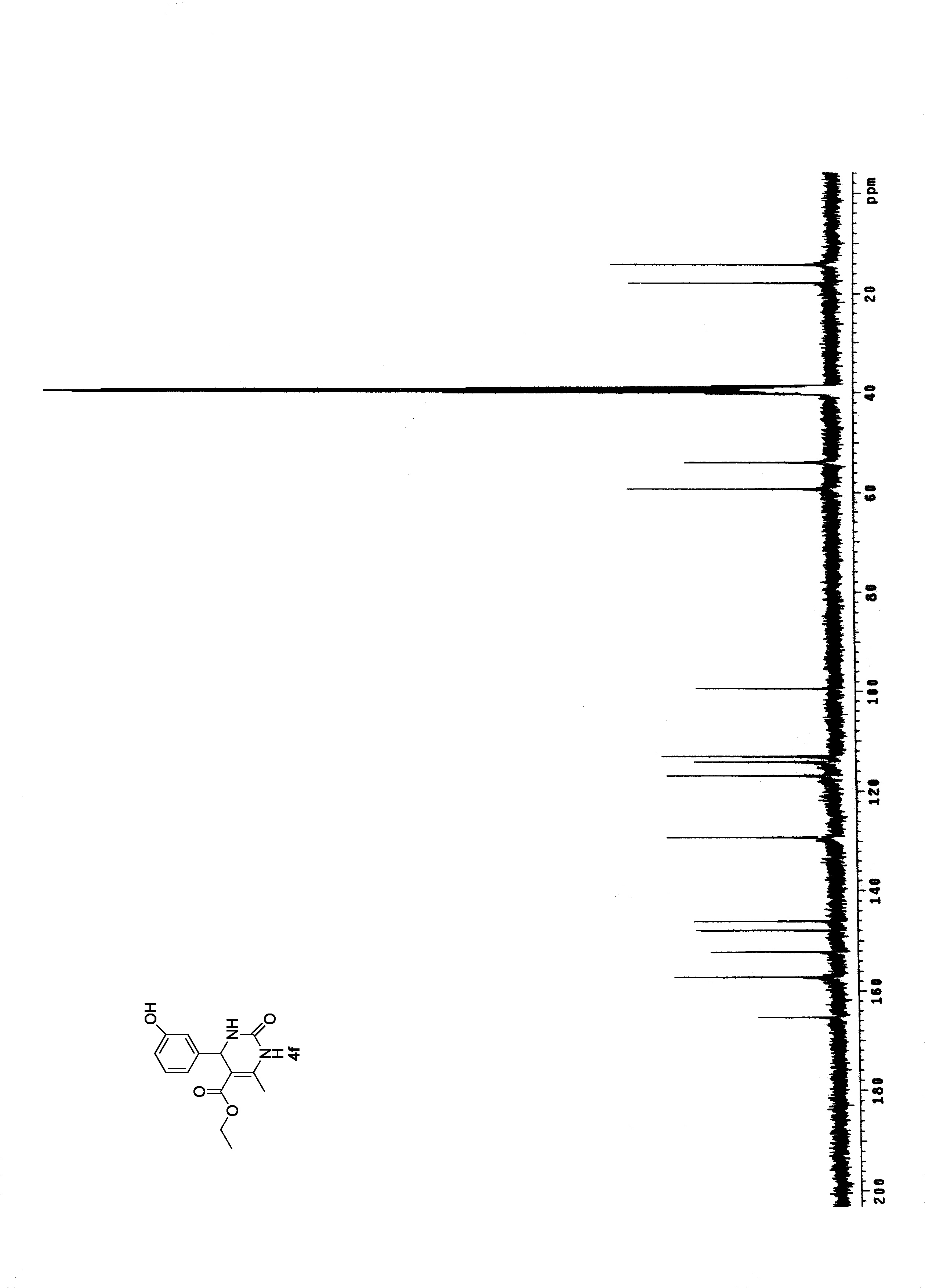


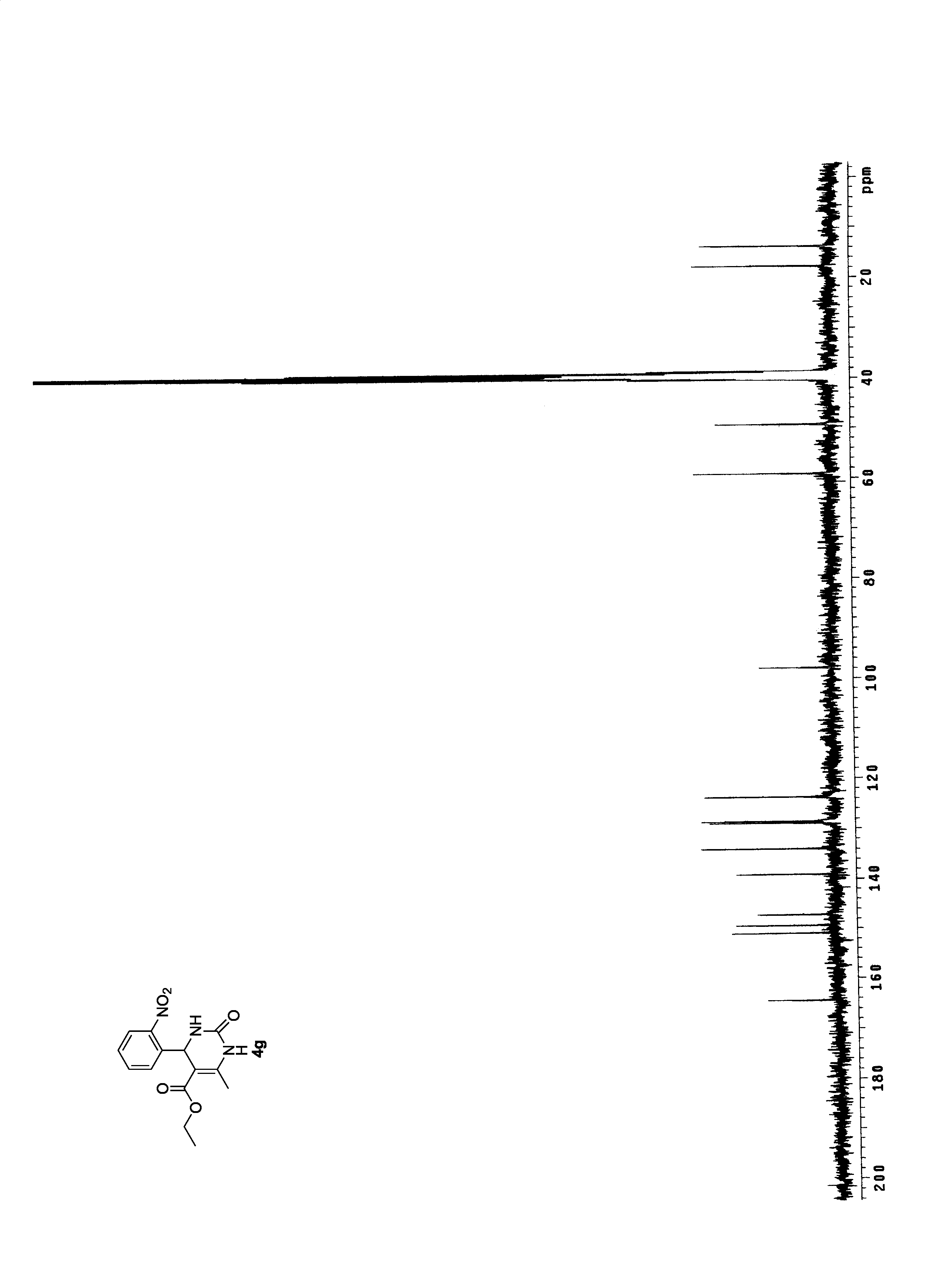


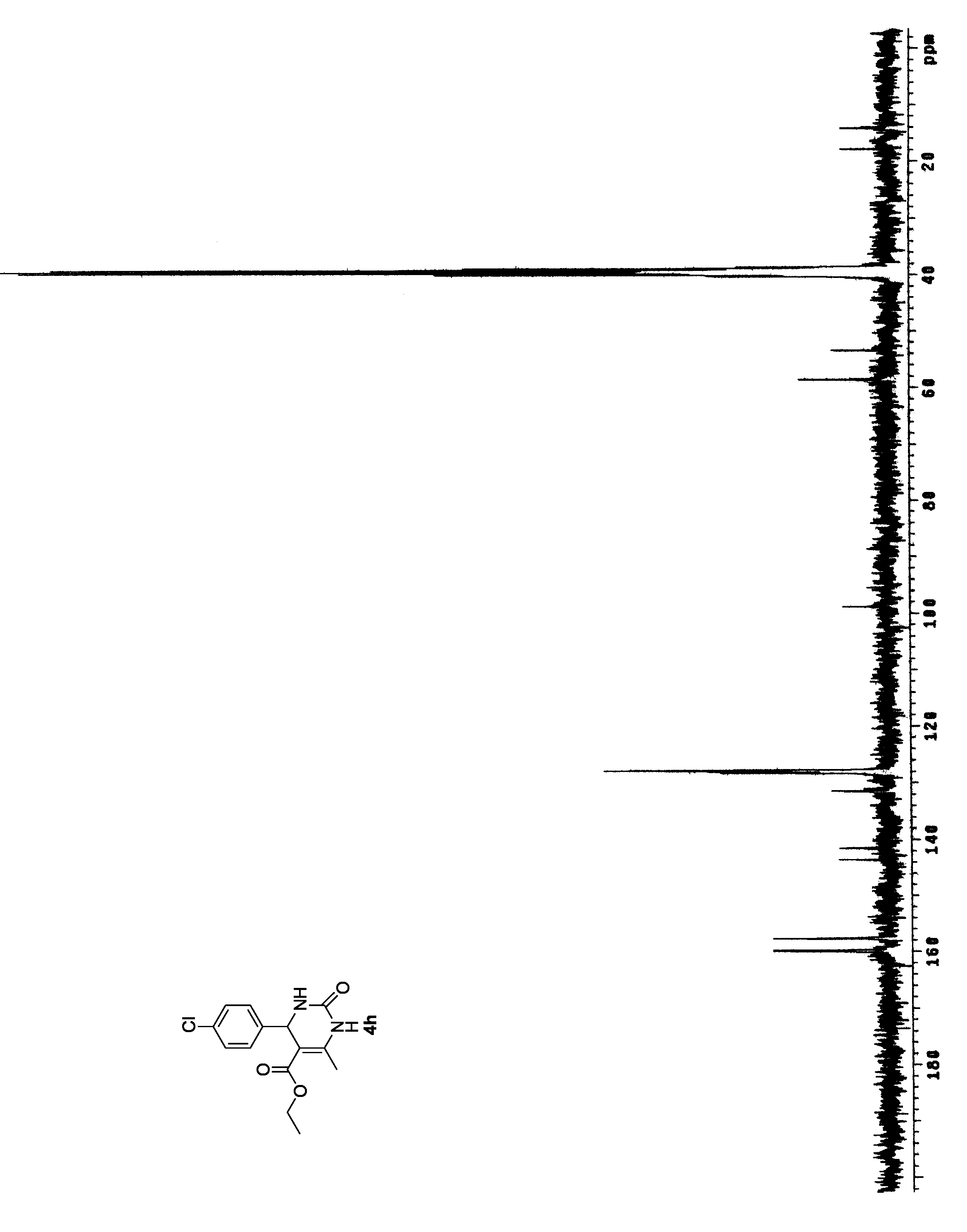


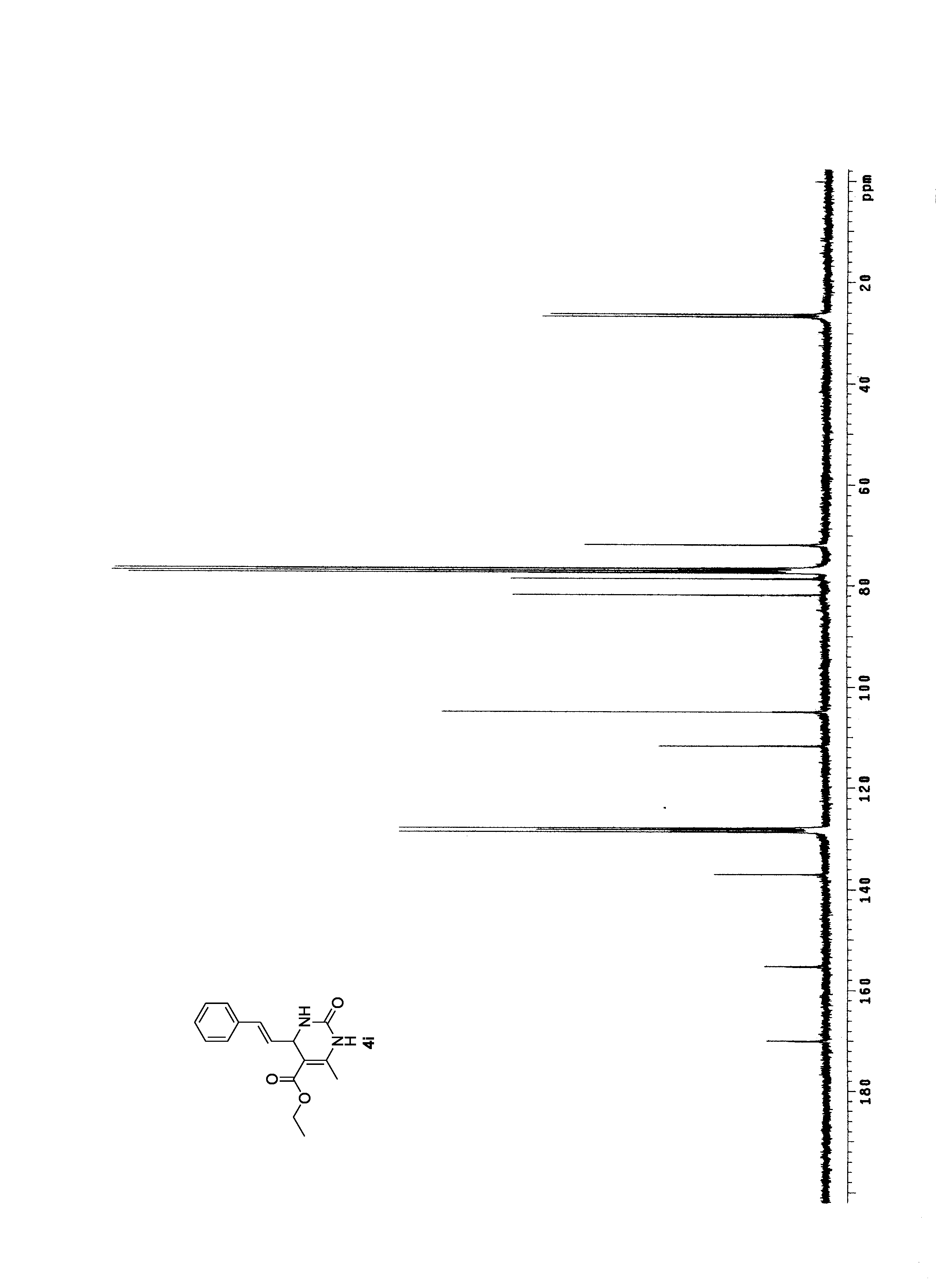


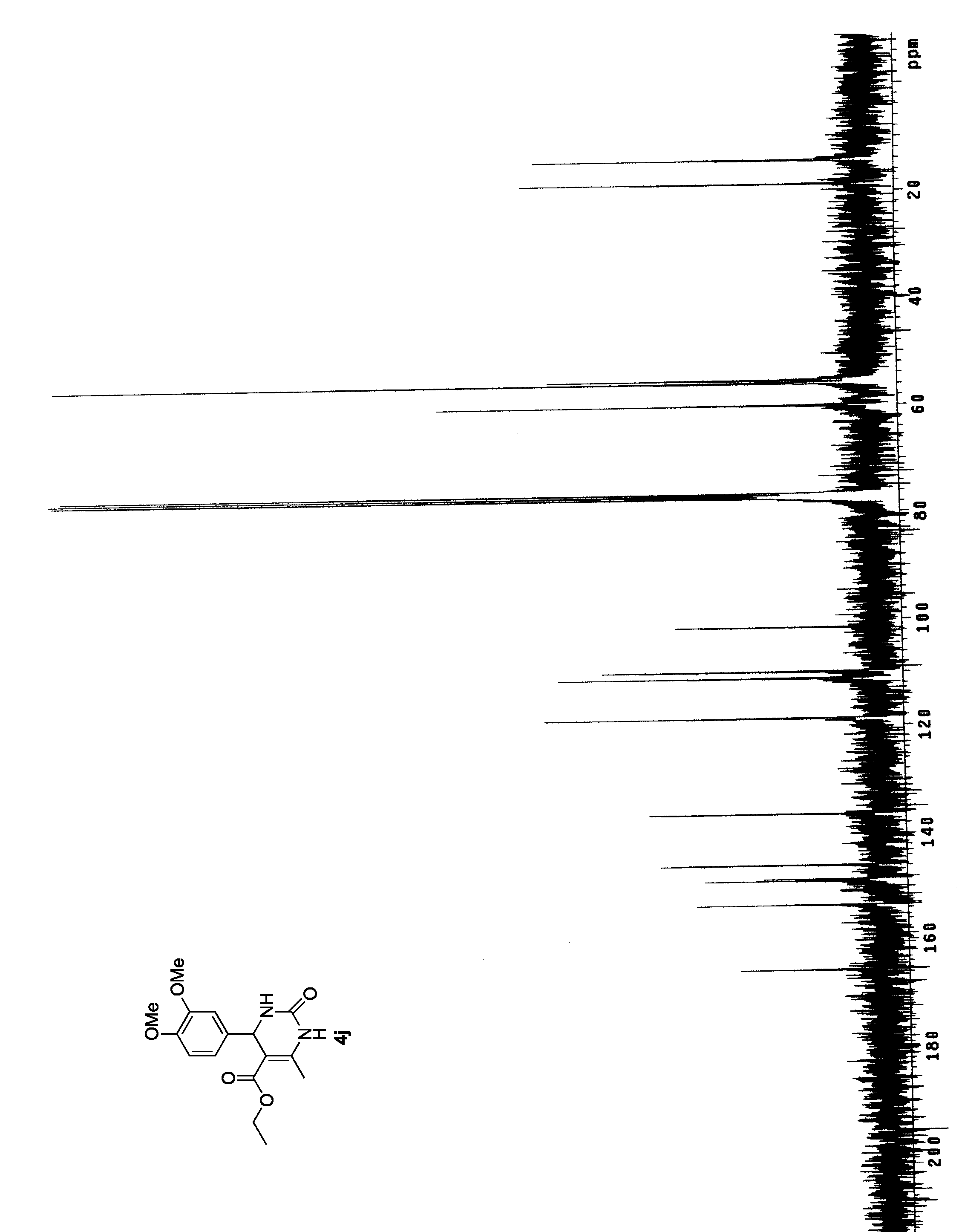


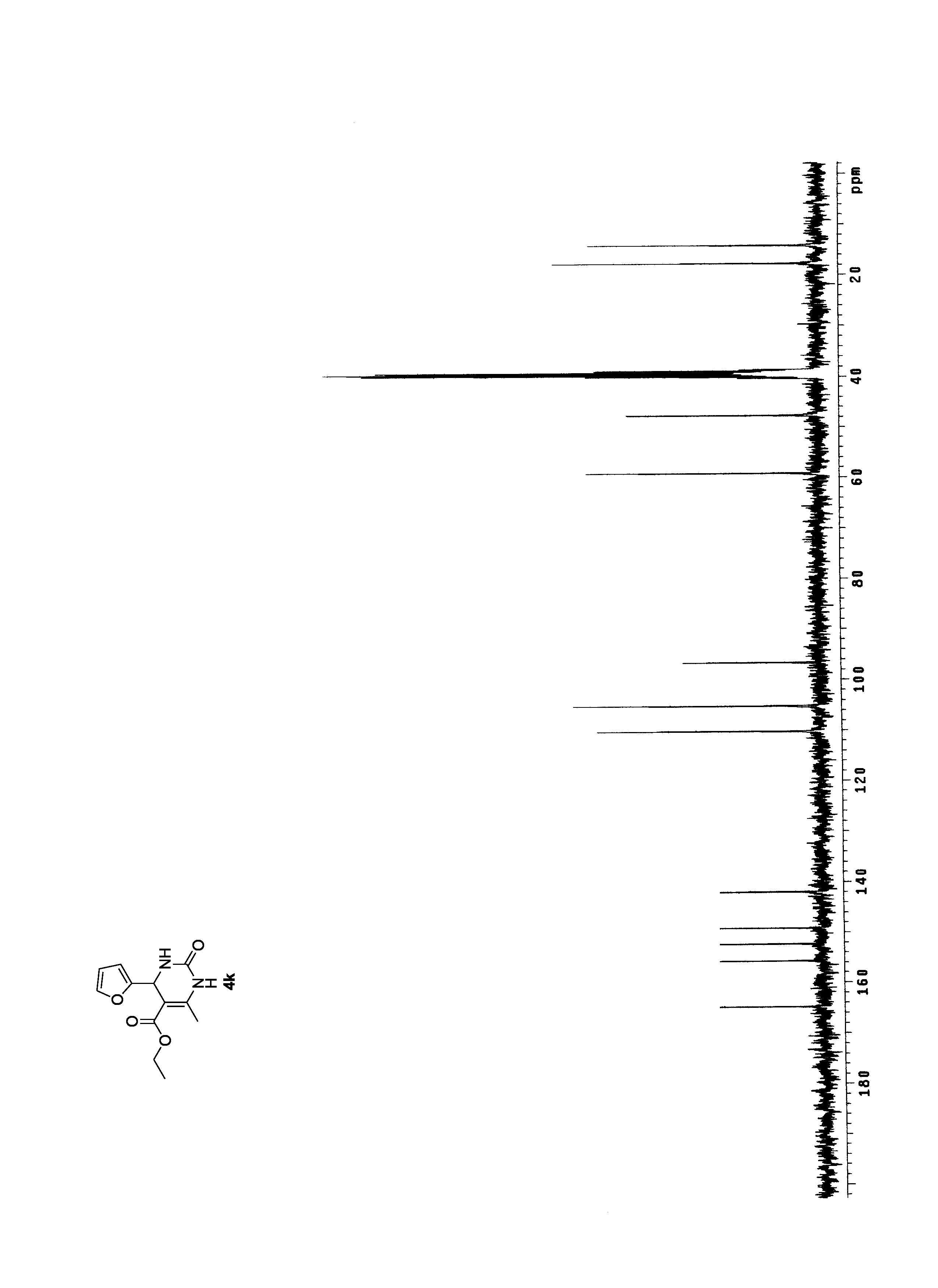


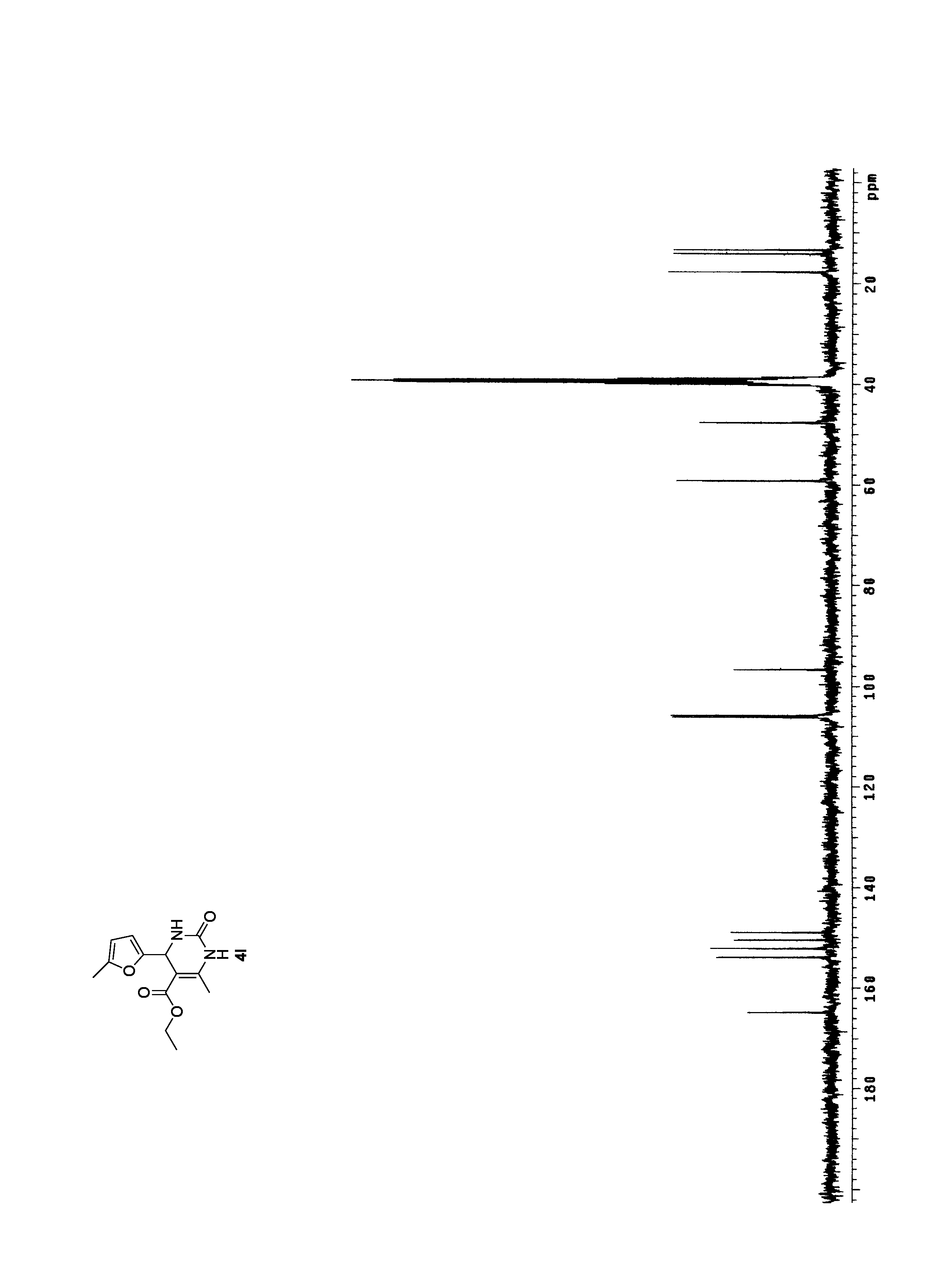


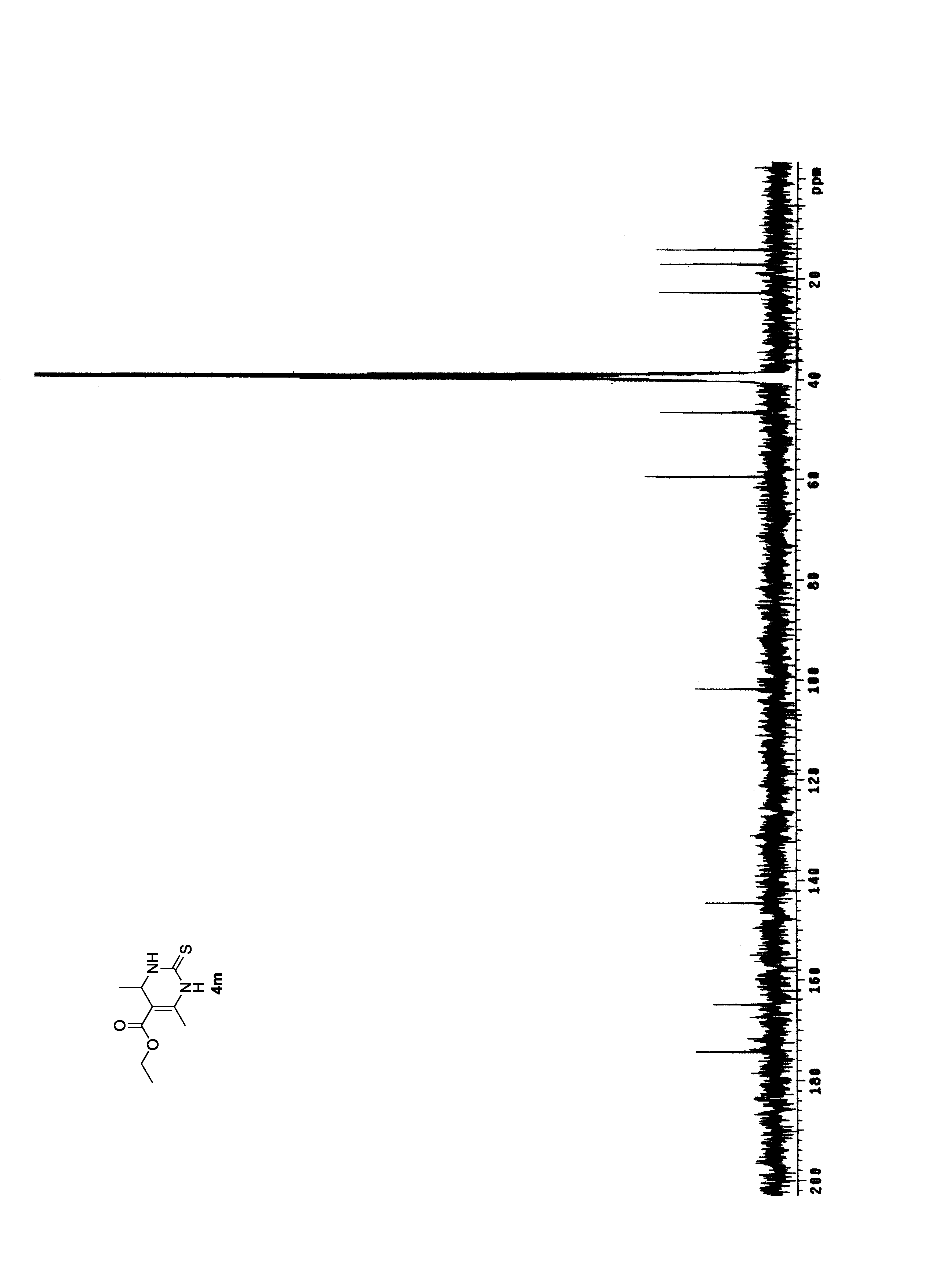


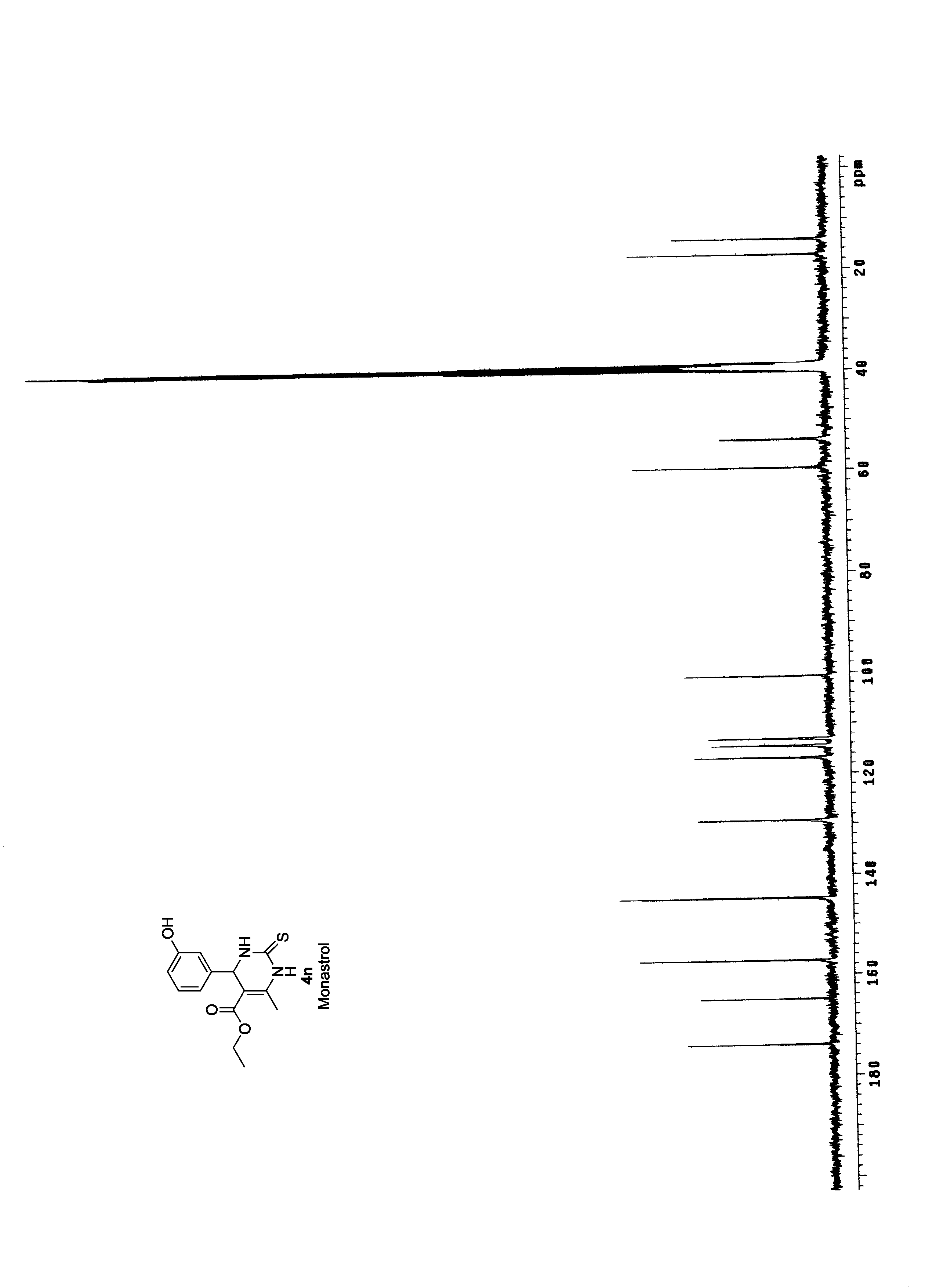


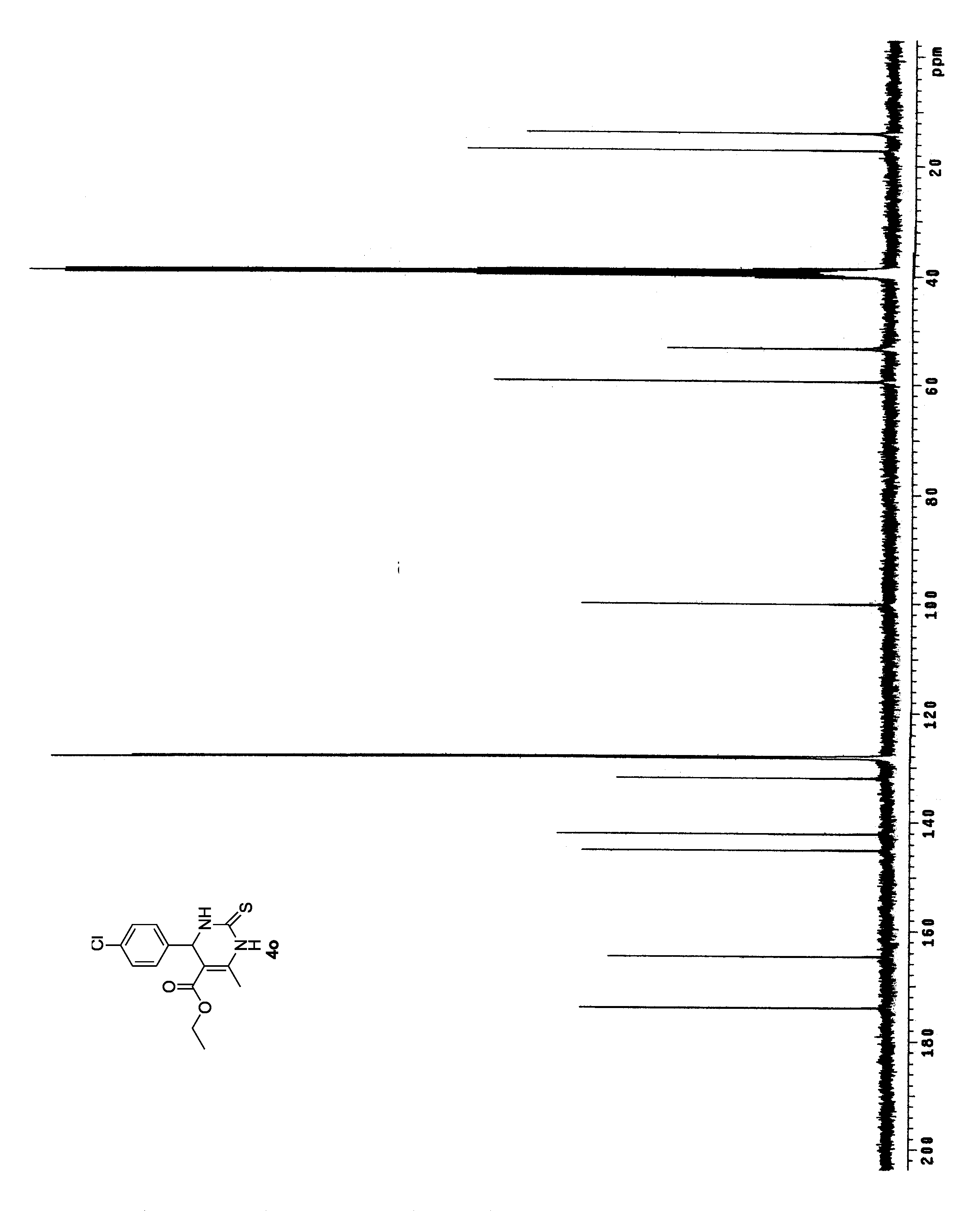


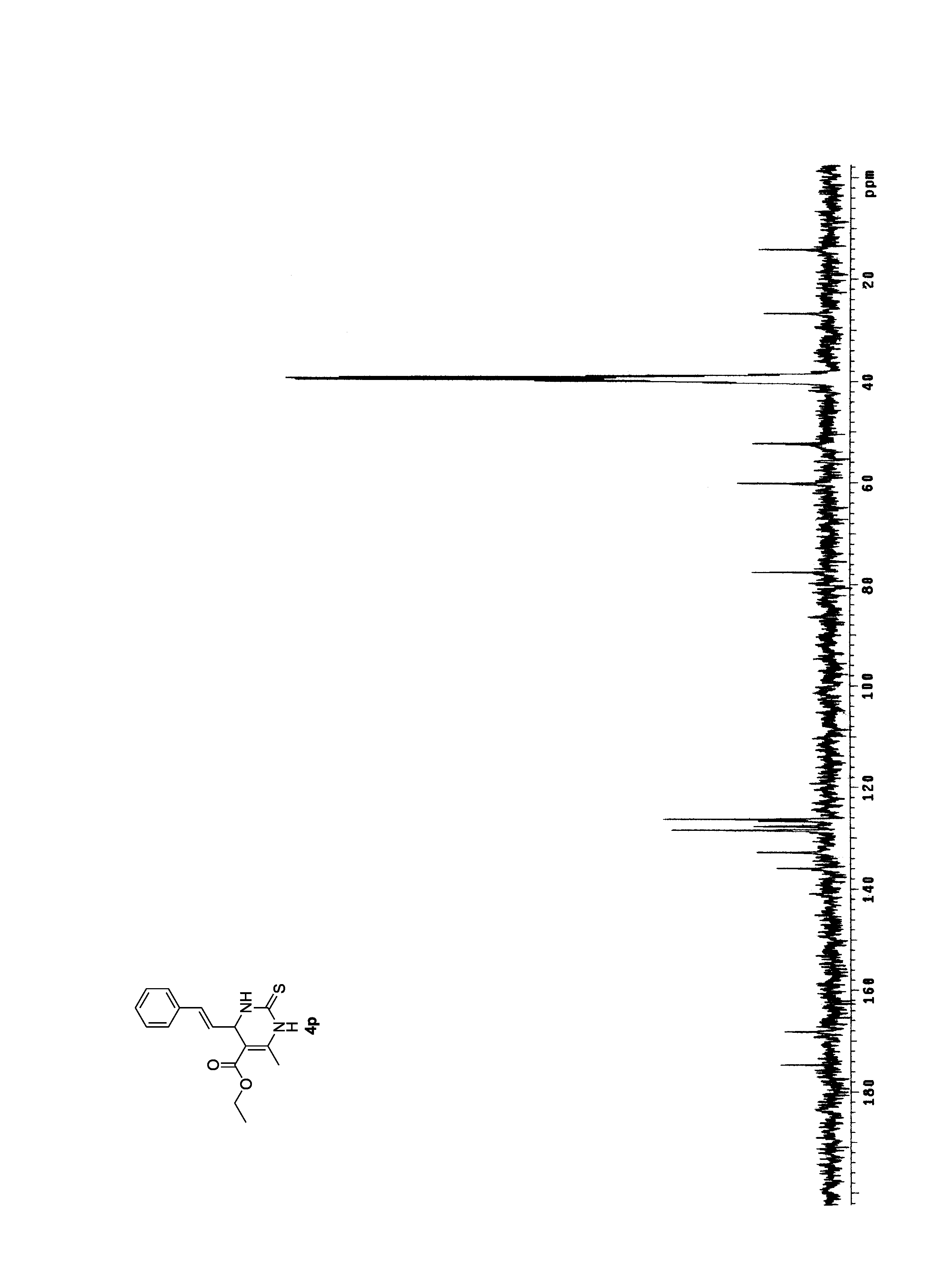


1. ^^ [↑](#endnote-ref-2)
