# Supplementary material for: Zeolite catalyzed solvent-free one-pot synthesis of dihydropyrimidin-2(1H)-ones – A practical synthesis of monastrol
Source: Beilstein J Org Chem. 2009 Feb 4;5:4. doi: 10.3762/bjoc.5.4 (PMC2649439; doi:10.3762/bjoc.5.4)
Supplement: File 2 — Experimental procedures for compounds 4a–4p [file Beilstein_J_Org_Chem-05-04-s002.doc]

# Zeolite catalyzed solvent-free one-pot synthesis of dihydropyrimidin-2(1*H*)-ones – A practical synthesis of monastrol

Mukund G. Kulkarni[[1]](#endnote-2)*, Sanjay W. Chavhan, Mahadev P. Shinde, Dnyaneshwar D. Gaikwad, Ajit S. Borhade, Attrimuni P. Dhondge, Yunnus B. Shaikh, Vijay B. Ningdale, Mayur P. Desai and Deekshaputra R. Birhade

Address: Department of Chemistry, University of Pune, Ganeshkhind, Pune 411007, Maharashtra, India

Email: Mukund G. Kulkarni - mgkulkarni@chem.unipune.ernet.in

* Corresponding author

Supporting Information

**Experimental procedures for compounds 4a**–**4p**

**5-Ethoxycarbonyl-4-phenyl-6-methyl-3,4-dihydropyrimidin-2-(1*H*)-one (4a)**

1.20 g, yield 98%

A mixture of benzaldehyde (0.50 g, 4.71 mmol), ethyl acetoacetate (0.613 g, 4.71mmol), urea (0.424 g, 7.06 mmol), and catalyst TS-1 (0.01 g, 2 wt % in relation to amount of benzaldehyde used) was heated at 50 °C for 10 min (TLC check). The reaction mixture after cooling to room temperature was poured into crushed ice and stirred for 5–10 min The solid separated was filtered and washed with ice-cold water. To separate the catalyst from the product, the mixture was treated with hot ethanol and filtered. The residue, being the catalyst, was dried and reused. The filtrate on concentration afforded the product, which was found to be sufficiently pure to obtain analytical data.

**4-Methyl-5-ethoxycarbonyl-6-methyl-3,4-dihydropyrimidin-2(1*H*)-one (4b)**

2.10 g, yield 93%,

A mixture of acetaldehyde (0.50 g, 11.3636 mmol), ethyl acetoacetate (1.478 g, 11.36 mmol), urea (1.022 g, 17.04 mmol), and catalyst TS-1 (0.01 g, 2 wt % in relation to amount of acetaldehyde used) was heated at 50 °C for 10 min (TLC check). The reaction mixture after cooling to room temperature was poured into crushed ice and stirred for 5–10 min The solid separated was filtered and washed with ice-cold water. To separate the catalyst from the product, the mixture was treated with hot ethanol and filtered. The residue, being the catalyst, was dried and reused. The filtrate on concentration afforded the product, which was found to be sufficiently pure to obtain analytical data.

**4-Ethyl-5-ethoxycarbonyl-6-methyl-3,4-dihydropyrimidin-2(1*H*)-one (4c)**

1.77 g, yield 97%

A mixture of propionaldehyde (0.50 g, 8.6206 mmol), ethyl acetoacetate (1.121g, 8.6206 mmol), urea (0.775 g, 12.93 mmol), and catalyst TS-1 (0.01 g, 2 wt % in relation to amount of propionaldehyde used) was heated at 50 °C for 12 min (TLC check). The reaction mixture after cooling to room temperature was poured into crushed ice and stirred for 5–10 min The solid separated was filtered and washed with ice-cold water. To separate the catalyst from the product, the mixture was treated with hot ethanol and filtered. The residue, being the catalyst, was dried and reused. The filtrate on concentration afforded the product, which was found to be sufficiently pure to obtain analytical data.

**4-Propyl-5-ethoxycarbonyl-6-methyl-3,4-dihydropyrimidin-2(1*H*)-one (4d)**

1.52 g, yield 97%

A mixture of butyraldehyde (0.50 g, 6.9444 mmol), ethyl acetoacetate (0.903 g, 6.94 mmol), urea (0.624 g, 10.41 mmol), and catalyst TS-1 (0.01g, 2 wt % in relation to amount of butyraldehyde used) was heated at 50 °C for15 min (TLC check). The reaction mixture after cooling to room temperature was poured into crushed ice and stirred for 5–10 min The solid separated was filtered and washed with ice-cold water. To separate the catalyst from the product, the mixture was treated with hot ethanol and filtered. The residue, being the catalyst, was dried and reused. The filtrate on concentration afforded the product, which was found to be sufficiently pure to obtain analytical data.

**5-Ethoxycarbonyl-4-(4-hydroxyphenyl)-6-methyl-3,4-dihydropyrimidin-2(1*H*)-one (4e)**

1.06 g, yield 94%

A mixture of 4-hydroxybenzaldehyde (0.50 g, 4.09 mmol), ethyl acetoacetate (0.532 g, 4.0929 mmol), urea (0.368 g, 6.14 mmol), and catalyst TS-1(0.01 g, 2 wt % in relation to amount of 4-hydroxybenzaldehyde used) was heated at 50 °C for 30 min (TLC check). The reaction mixture after cooling to room temperature was poured into crushed ice and stirred for 5–10 min The solid separated was filtered and washed with ice-cold water. To separate the catalyst from the product, the mixture was treated with hot ethanol and filtered. The residue, being the catalyst, was dried and reused. The filtrate on concentration afforded the product, which was found to be sufficiently pure to obtain analytical data.

**5-Ethoxycarbonyl-4-(3-hydroxyphenyl)-6-methyl-3,4-dihydropyrimidin-2(1*H*)-one (4f)**

1.07 g, yield 95%

A mixture of 3-hydroxybenzaldehyde (0.50 g, 4.09 mmol), ethyl acetoacetate (0.532 g, 4.09 mmol), urea (0.368 g, 6.14 mmol), and catalyst TS-1 (0.01g, 2 wt % in relation to amount of 3-hydroxybenzaldehyde used) was heated at 50 °C for 25 min (TLC check). The reaction mixture after cooling to room temperature was poured into crushed ice and stirred for 5–10 min The solid separated was filtered and washed with ice-cold water. To separate the catalyst from the product, the mixture was treated with hot ethanol and filtered. The residue, being the catalyst, was dried and reused. The filtrate on concentration afforded the product, which was found to be sufficiently pure to obtain analytical data.

**5-Ethoxycarbonyl-4-(2-nitrophenyl)-6-methyl-3,4-dihydropyrimidin-2(1*H*)-one (4g)**

0.90 g, yield 90%

A mixture of 2-nitrobenzaldehyde (0.50 g, 3.31 mmol), ethyl acetoacetate (0.430 g, 3.31 mmol), urea (0.298 g, 4.97 mmol), and catalyst TS-1 (0.01 g, 2 wt % in relation to amount of 2-nitrobenzaldehyde used) was heated at 50 °C for 35 min (TLC check). The reaction mixture after cooling to room temperature was poured into crushed ice and stirred for 5–10 min The solid separated was filtered and washed with ice-cold water. To separate the catalyst from the product, the mixture was treated with hot ethanol and filtered. The residue, being the catalyst, was dried and reused. The filtrate on concentration afforded the product, which was found to be sufficiently pure to obtain analytical data.

**5-Ethoxycarbonyl-4-(4-chlorophenyl)-6-methyl-3,4-dihydropyrimidin-2(1*H*)-one (4h)**

1.01 g, yield 96%

A mixture of 4-chlorobenzaldehyde (0.50 g, 3.56 mmol), ethyl acetoacetate (0.463 g, 3.56 mmol), urea (0.320 g, 5.34 mmol), and catalyst TS-1 (0.01 g, 2 wt % in relation to amount of 4-chlorobenzaldehyde used) was heated at 50 °C for 20 min (TLC check). The reaction mixture after cooling to room temperature was poured into crushed ice and stirred for 5–10 min The solid separated was filtered and washed with ice-cold water. To separate the catalyst from the product, the mixture was treated with hot ethanol and filtered. The residue, being the catalyst, was dried and reused. The filtrate on concentration afforded the product, which was found to be sufficiently pure to obtain analytical data.

**5-Ethoxycarbonyl-6-methyl-4-styryl-3,4-dihydropyrimidin-2(1*H*)-one (4i)**

1.04g, yield 96%

A mixture of cinnamic aldehyde (0.50 g, 3.78 mmol), ethyl acetoacetate (0.492g, 3.78 mmol), urea (0.340 g, 5.67 mmol), and catalyst TS-1 (0.01 g, 2 wt % in relation to amount of cinnamic aldehyde used) was heated at 50 °C for 10 min (TLC check). The reaction mixture after cooling to room temperature was poured into crushed ice and stirred for 5–10 min The solid separated was filtered and washed with ice-cold water. To separate the catalyst from the product, the mixture was treated with hot ethanol and filtered. The residue, being the catalyst, was dried and reused. The filtrate on concentration afforded the product, which was found to be sufficiently pure to obtain analytical data.

**5-Ethoxycarbonyl-4-(3,4-dimethoxyphenyl)-6-methyl-3,4-dihydropyrimidin-2(1*H*)-one (4j)**

0.92 g, yield 96%

A mixture of 3,4-dimethoxybenzaldehyde (0.50 g, 3.01 mmol), ethyl acetoacetate (0.391 g, 3.01 mmol), urea (0.271 g, 4.52 mmol), and catalyst TS-1(0.01 g, 2 wt % in relation to amount of 3,4-dimethoxybenzaldehyde used) was heated at 50 °C for 18 min (TLC check). The reaction mixture after cooling to room temperature was poured into crushed ice and stirred for 5–10 min The solid separated was filtered and washed with ice-cold water. To separate the catalyst from the product, the mixture was treated with hot ethanol and filtered. The residue, being the catalyst, was dried and reused. The filtrate on concentration afforded the product, which was found to be sufficiently pure to obtain analytical data.

5-Ethoxycarbonyl-4-(2-furyl)-6-methyl-3,4-dihydropyrimidin-2(1*H*)-one (4k)

1.24 g, yield 95%

A mixture of furfural (0.50 g, 5.20 mmol), ethyl acetoacetate (0.676 g, 5.20 mmol), urea (0.468 g, 7.80 mmol), and catalyst TS-1 (0.01 g, 2 wt % in relation to amount of furfural used) was heated at 50 °C for 12 min (TLC check). The reaction mixture after cooling to room temperature was poured into crushed ice and stirred for 5–10 min The solid separated was filtered and washed with ice-cold water. To separate the catalyst from the product, the mixture was treated with hot ethanol and filtered. The residue, being the catalyst, was dried and reused. The filtrate on concentration afforded the product, which was found to be sufficiently pure to obtain analytical data.

**5-Ethoxycarbonyl-4-(5-methyl-2-furyl)-6-methyl-3,4-dihydropyrimidin-2(1*H*)-one (4l)**

1.15 g, yield 95%

A mixture of 5-methylfurfural (0.50 g, 4.58 mmol), ethyl acetoacetate (0.596 g, 4.58 mmol), urea (0.412 g, 6.8706 mmol), and catalyst TS-1 (0.01 g, 2 wt % in relation to amount of 5-methylfurfural used) was heated at 50 °C for 15 min (TLC check). The reaction mixture after cooling to room temperature was poured into crushed ice and stirred for 5–10 min The solid separated was filtered and washed with ice-cold water. To separate the catalyst from the product, the mixture was treated with hot ethanol and filtered. The residue, being the catalyst, was dried and reused. The filtrate on concentration afforded the product, which was found to be sufficiently pure to obtain analytical data.

**5-ethoxycarbonyl-4,6-dimethyl-3,4-dihydropyrimidin-2(1*H*)-thione (4m)**

2.30 g, yield 95%

A mixture of acetaldehyde (0.50 g, 11.36 mmol), ethyl acetoacetate (1.478 g, 11.36 mmol), thiourea (1.227 g, 17.05 mmol), and catalyst TS-1 (0.01 g, 2 wt % in relation to amount of acetaldehyde used) was heated at 50 °C for 20 min (TLC check). The reaction mixture after cooling to room temperature was poured into crushed ice and stirred for 5–10 min The solid separated was filtered and washed with ice-cold water. To separate the catalyst from the product, the mixture was treated with hot ethanol and filtered. The residue, being the catalyst, was dried and reused. The filtrate on concentration afforded the product, which was found to be sufficiently pure to obtain analytical data.

**5-Ethoxycarbonyl-4-(3-hydroxyphenyl)-6-methyl-3,4-dihydropyrimidin-2(1*H*)-thione (4n)**

1.13 g, yield 94%

A mixture of 3-hydroxybenzaldehyde (0.50 g, 4.09 mmol), ethyl acetoacetate (0.532 g, 4.09 mmol), thiourea (0.442 g, 6.14 mmol), and catalyst TS-1 (0.01 g, 2 wt % in relation to amount of 3-hydroxybenzaldehyde used) was heated at 50 °C for 33 min (TLC check). The reaction mixture after cooling to room temperature was poured into crushed ice and stirred for 5–10 min The solid separated was filtered and washed with ice-cold water. To separate the catalyst from the product, the mixture was treated with hot ethanol and filtered. The residue, being the catalyst, was dried and reused. The filtrate on concentration afforded the product, which was found to be sufficiently pure to obtain analytical data.

**5-Ethoxycarbonyl-4-(4-chlorophenyl)-6-methyl-3,4-dihydropyrimidin-2(1*H*)-thione (4o)**

1.05 g, yield 95%

A mixture of 4-chlorobenzaldehyde (0.50 g, 3.56 mmol), ethyl acetoacetate (0.463 g, 3.56 mmol), thiourea (0.384 g, 5.34 mmol), and catalyst TS-1 (0.01 g, 2 wt % in relation to amount of 4-chlorobenzaldehyde used) was heated at 50 °C for 20 min (TLC check). The reaction mixture after cooling to room temperature was poured into crushed ice and stirred for 5–10 min The solid separated was filtered and washed with ice-cold water. To separate the catalyst from the product, the mixture was treated with hot ethanol and filtered. The residue, being the catalyst, was dried and reused. The filtrate on concentration afforded the product, which was found to be sufficiently pure to obtain analytical data.

**5-Ethoxycarbonyl-6-methyl-4-styryl-3,4-dihydropyrimidin-2(1*H*)-thione (4p)**

1.08 g, yield 95%

A mixture of cinnamic aldehyde (0.50 g, 3.78 mmol), ethyl acetoacetate (0.492 g, 3.78 mmol), thiourea (0.408 g, 5.67 mmol), and catalyst TS-1 (0.01 g, 2 wt % in relation to amount of cinnamic aldehyde used) was heated at 50 °C for 14 min (TLC check). The reaction mixture after cooling to room temperature was poured into crushed ice and stirred for 5–10 min The solid separated was filtered and washed with ice-cold water. To separate the catalyst from the product, the mixture was treated with hot ethanol and filtered. The residue, being the catalyst, was dried and reused. The filtrate on concentration afforded the product, which was found to be sufficiently pure to obtain analytical data.

1. ^^ [↑](#endnote-ref-2)
